# Depth-Imaging for Gait Analysis on a Treadmill in Older Adults at Risk of Falling

**DOI:** 10.1109/JTEHM.2023.3277890

**Published:** 2023-05-19

**Authors:** Michel Hackbarth, Jessica Koschate, Sandra Lau, Tania Zieschang

**Affiliations:** School of Medicine and Health ScienceDepartment for Health Services Research, Geriatrics DivisionCarl von Ossietzky University Oldenburg 26129 Oldenburg Germany

**Keywords:** Gait, older people, falls, depth-camera

## Abstract

Background: Accidental falls are a major health issue in older people. One significant and potentially modifiable risk factor is reduced gait stability. Clinicians do not have sophisticated kinematic options to measure this risk factor with simple and affordable systems. Depth-imaging with AI-pose estimation can be used for gait analysis in young healthy adults. However, is it applicable for measuring gait in older adults at a risk of falling? Methods: In this methodological comparison 59 older adults with and without a history of falls walked on a treadmill while their gait pattern was recorded with multiple inertial measurement units and with an Azure Kinect depth-camera. Spatiotemporal gait parameters of both systems were compared for convergent validity and with a Bland-Altman plot. Results: Correlation between systems for stride length (r=.992, $\text{p} < 0.001$) and stride time (r=0.914, $\text{p} < 0.001$) was high. Bland-Altman plots revealed a moderate agreement in stride length (−0.74 ± 3.68 cm; [−7.96 cm to 6.47 cm]) and stride time (−3.7±54 ms; [−109 ms to 102 ms]). Conclusion: Gait parameters in older adults with and without a history of falls can be measured with inertial measurement units and Azure Kinect cameras. Affordable and small depth-cameras agree with IMUs for gait analysis in older adults with and without an increased risk of falling. However, tolerable accuracy is limited to the average over multiple steps of spatiotemporal parameters derived from the initial foot contact. Clinical Translation Statement— Gait parameters in older adults with and without a history of falls can be measured with inertial measurement units and Azure Kinect. Affordable and small depth-cameras, developed for various purposes in research and industry, agree with IMUs in clinical gait analysis in older adults with and without an increased risk of falling. However, tolerable accuracy to assess function or monitor changes in gait is limited to the average over multiple steps of spatiotemporal parameters derived from the initial foot contact.

## I. Introduction

Reduced gait stability in older adults is a risk factor for falls and is associated with loss of independent living, disability, and lower quality of life. Especially people with a history of falls have a higher risk of falling again [1]. It has been proposed that gait changes can be used as a marker of fall risk and deterioration of functional capabilities [2]. In contrast to obvious gait instabilities, subclinical changes in gait parameters and dynamic gait stability (e.g. margin of stability, MoS) cannot be reliably quantified by visual observations. Advanced technology in gait analysis has overcome this barrier with accurate motion capture systems [3]. Infrared-cameras, as a gold standard of motion-capture systems, track reflective marker-sets on a patient’s body and measure kinematics with high accuracy and a high sample rate. However, these systems are cost intensive, consist of multiple cameras, require a calibration procedure and often a complex marker-set that needs to be attached to the subject. Therefore, this technique is limited to the laboratory setting. Inertial measurement units (IMU) and joint-tracking from depth-images emerged over the past years as promising alternatives for simple gait analysis. Especially IMU-systems are widely available for gait analysis but limited to measurement of acceleration and angular velocity at the point of attachment and therefore not able to calculate spatial relations between body parts (e.g. step width, margin of stability). Further, depth-imaging cameras can be used to model human movement, while often, only a single camera is required [4]. One of these depth-cameras is the commercially available Microsoft Azure Kinect (AK). These novel cameras are a spatial computing developer kit for various areas of application as manufacturing, healthcare, media, robotics and retail. Like its predecessor the Kinect v2, the AK is operating with the Time-of-Flight principle but with a higher resolution and the most recent version of a pose estimation AI known as Bodytracking SDK [5]. A system of depth-camera and body tracking could help to implement monitoring tools from the lab in the daily care of older patients – a translational process necessary to evaluate and re-assess individual falls prevention interventions for populations with declining functional abilities, such as gait. The comparison of AK to the gold standard of a marker-based motion capture system indicated temporal accuracy for the calculation of spatiotemporal gait-parameters in healthy young adults with an average error of −26 ± 22 ms for the correct identification of the initial foot contact, and −88 ± 34 ms for the terminal foot contact during treadmill walking with significantly higher spatial accuracy in feet-tracking than the predecessor Kinect v2 [6]. Older adults are walking with increased cadence and shorter stride length on a treadmill in comparison to over ground walking [7]. Despite the lack of an effective treadmill familiarization for older adults [8] the advantages of patient safety with a fall harness and the ability of capturing consecutive gait cycles in a small space without external influences makes gait analysis in a clinical context more feasible [7]. Dynamic gait-stability as a relevant indicator of fall risk, can be quantified by the MoS as a relation of the centre of mass to the base of support (BoS) with an inverted pendulum model [9]. Therefore, calculation of the MoS requires the tracking of a velocity adjusted centre of mass. In clinical research, the centre of mass is often approximated with fixed anatomical positions, as the midpoint of the pelvis, which can be tracked by depth cameras [10]. Mehdizadeh et al. [11] for example used the MoS from AK in a care facility setting in older people with dementia to estimate the risk of falling. Gait stability is also affected by the arm swing amplitude [12] which can be assessed with wrist worn IMUs [13] as well as tracking the wrists with AK. In the current state, gait analysis with AK was mainly evaluated in younger adults and populations without a history of falls [6], [14], [15]. Older adults with and without a risk of falling potentially show differences in their gait-pattern which could have an impact on gait analysis systems. Normal aging effects including alteration of the gait pattern have been identified as risk factors for falling in older adults [16]. Older adults with a history of falls have an additional, on average 3-fold increased risk of falling [17]. Especially slower gait speed and short step- and stride lengths were reported consistently as gait characteristics of older people with a history of falls [18], and/or fear of falling (FoF) [19]. Changes in quantitative gait parameters, such as of older adults can be used as independent fall risk markers [20]. Verghese and colleagues showed that a slower gait speed (−10cm/s), shorter stride length (−10cm), shorter swing phase (−10%), longer double support phase (+10%), higher stride length variability (+10%) and higher swing time variability (+10%) are all associated with a significantly higher risk-ratio for falling. The 10-unit changes were selected to make the observations clinically intuitive [20]. The observation of changes in gait, in response to rehabilitation are valuable for the assessment of efficacy of individual therapies. Changes in gait are likely to be observed in treadmill walking, despite the known systematical differences to over ground-walking [7]. Considering the marker-less principle, depth-cameras can be an alternative for motion analysis and fall risk estimation outside of high-tech gait laboratories, e.g. in clinical settings [21]. Therefore, it should be tested whether this new iteration of depth-cameras agree with other clinically used systems, as IMU’s, to measure gait parameters in older adults without a history of falls and also older adults with a history of falls, as a population with increased fall risk and whether AK is able to measure additional, gait stability specific measurements. In this study we conducted walking trials on a treadmill with an older population with and without a history of falls, and used a novel model of depth-cameras in comparison with multiple inertial measurement units to measure gait in a clinical setting. The aim is to test the agreement between AK and established IMU-networks in spatiotemporal gait parameters, related to fall risk of older people with and without a fall history.

## II. Methods

### A. Participants and Recruitment

The study was approved by the Medical Ethics Committee of the University of Oldenburg (Nr.:2019-133) and is listed in the German Clinical Trials Register (DRKS00020363). Prior to data collection, written informed consent was given by all participants. All procedures were conducted in accordance with the provisions of the Declaration of Helsinki. A power calculation on the basis of gait parameters from Roeles et al. [22] revealed a minimum of 50 participants. Participants were recruited via public announcements in newspapers, flyers and the online presence of the University of Oldenburg. From July 2020 until February 2022, N = 59 older adults ≥70 years of age with a history of falls (n=29) and without a history of falls (n=30) were recruited. Requirement for all participants was the ability to walk independently. Participants with a body mass above 135 kg, body height above 185 cm (technical limitations), neurological and orthopedic disorders, that have an acute effect on the gait pattern, severe arthritis, joint replacement surgery in the past 6 months, blindness, and inability to provide informed consent were excluded from the trial. The participants filled in questionnaires for anthropometric- and health related data, including walking-, hearing-aids, glasses, history of falls in the last 12 months as well as the Falls Efficacy Scale-International (FES-I).

### B. Experimental Setup

**FIGURE 1.**
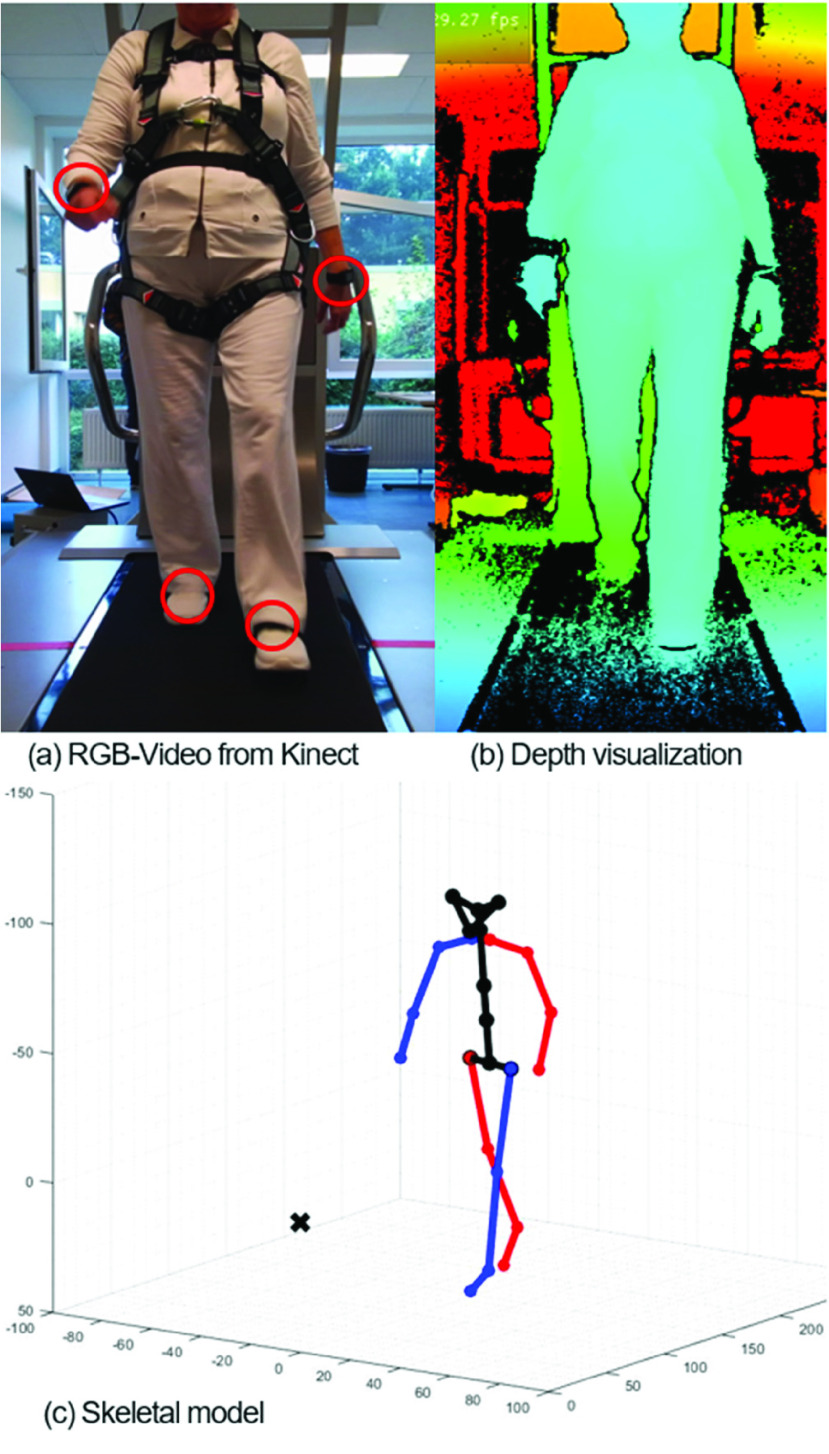
(a) RGB-video from Kinect camera. Red circles show attachment of IMU’s. (b) Visualization of depth-measurement from blue (near objects) to red (far away objects). (c) Skeletal model from pose estimation-AI of a depth-video. The black cross marks the camera position in relation to the subject.

The experiments were conducted at the Rehabilitation Centre Oldenburg in Oldenburg, Germany. All participants walked on a treadmill (BalanceTutor, MediTouch, Netanyha, Israel) at their individually preferred walking speed. Participants wore a harness, connected to an overhead suspension system to ensure safety during the walking trials. Participants were equipped with six inertial measurement units (OPAL, APDM, Portland, USA) on the feet, wrists, at the lumbar level and the sternum. Each of the six IMUs recorded 3-axis acceleration and angular velocity at a sample rate of 128 Hz. The treadmill logged the applied belt-speed at a sample rate of 64 Hz. The participants were recorded with an infrared-depth-camera (Azure Kinect DK, Microsoft, Redmond, USA), which provides 3D-kinematics, using the time-of-flight principle to calculate depth [5]. Data was recorded with a sample rate of 30 Hz at the highest depth-resolution of $640\times 576$ pixels and an according RGB-resolution of $2048\times 1536$ pixels [5]. The Azure Kinect Recorder from the Azure Kinect Software Developer Kit (Version 1.4.1, Microsoft, Redmond, USA) was used for data recording. The camera was set up in front of the treadmill at a height of 90 cm above the ground providing a full body image with the lower extremities centred. The distance from the lens to the longitudinal midpoint of the treadmill belt was approximately 150 cm [23] and the camera angle relative to the longitudinal axis of the subject at 0° (Figure 1a, 1b) to ensure pose estimation for the right and left extremities without the visual occlusion of one side [23], [24]. The camera was aligned horizontally to the ground via an integrated acceleration sensor. At least 60 minutes prior to the measurement, the camera was preheated to reduce the standard deviation of depth-sensing [23]. Systems were not synchronized on step-by-step basis, since the analysis focuses on average parameters of the same time interval of 120 seconds until the treadmill came to a stop and the participants stood still.

### C. Data Collection

In order to ensure a reference pose for the AK and the IMU’s, the subjects were instructed to stand still during the first and last 3 seconds of the measurement. Each subject completed one trial with approximately 5 minutes of walking. The first 3 minutes served to find the preferred walking speed and to fundamentally familiarize with walking on the treadmill, followed by a two-minute period for gait analysis during walking at the chosen speed. Preferred walking speed on the treadmill was determined via feedback from the subjects as follows: the treadmill speed was increased and the participants gave feedback when the preferred walking speed was first reached and first exceeded. The average of these two speeds represents the preferred walking speed, similar to other studies [25], [26]. Raw inertial data, depth-videos and treadmill speed were stored for further signal processing and analysis.

### D. Signal Processing

The Azure Kinect Bodytracking Software Developer Kit Version 1.2.0 with a pose estimation algorithm via Direct ML was used to extract 3D-positions of 32 joints [5] from the depth-videos. The body-tracking results were saved as 3-axis coordinates in distance from the depth-lens of the camera. Bodytracking-results, IMU-data, and treadmill-speed were processed with a custom Matlab-script (Matlab R2022a, Mathworks Inc., California, USA). Acceleration and angular velocity from IMU’s were filtered with a $4^{th}$-order butterworth-filter with a cut-off frequency of 10 Hz and joint positions from the AK were filtered with a $1^{st}$-order butterworth-filter with a cut-off frequency of 6 Hz [27]. Non-walking parts at the beginning of the trial were cut with threshold-detections. The data from the time interval of finding the preferred walking speed and the familiarization period were discarded. Data from both systems of the last 120 seconds of walking in each trial were analysed. Arm swing amplitude in degree was calculated from angular velocity at the wrist for IMU [13] and for AK with the cosine theorem via the position of the shoulder, arm length and subsequent maximum positions of the wrist. To calculate gait-parameters, the events of initial contact and toe-off of the feet with the ground were identified with algorithms for 3D-kinematic data from AK [28] and inertial data from foot worn IMU’s [29]. The AK method is a position-based algorithm from Zeni et al. [28] and was also used in the mentioned AK validation study from Albert et al. [6]. The accuracy of the algorithm has been tested to a gold standard, with an identification of 94% of gait events with a 16 ms error [28]. The IMU method is an algorithm based on the angular velocity around the medio-lateral axis of the foot [29] and has been validated at 100 Hz with an average error of −2 ms (CI $-16\,\,12$ ms) for detecting the initial contact and average error of 35 ms for detecting the toe-off in comparison to foot switches at 200 Hz [30]. The gait parameters stride time (s) and stride length (m) were calculated from the initial contact for the AK and IMU system. For IMU-stride length, the anteposterior acceleration between toe off and following initial contacts of the same foot were double-integrated from acceleration over velocity to distance plus the treadmill translation during the stride. AK stride length was defined as the difference in anteposterior-distance from the leading ankle to the camera between consecutive initial contacts plus the treadmill translation during the stride. Single support time (s) and double support time (s) were calculated from the combination of the initial contact and toe-off. The MoS in mediolateral and anteposterior direction at each initial contact was calculated with an inverted pendulum model utilizing the velocity adjusted centre of mass in relation to the base of support [9] where the centre of mass is estimated as the midpoint of the pelvis and the base of support as the ankle and toe landmark of AK [5], [10]. MoS (cm) as well as step width (cm) could only be calculated from 3D-kinematic data of AK but were calculated regardless to utilize the methods up to their potential. All steps during the 120 seconds window were averaged for statistical analysis.

### E. Statistical Analysis

The statistical analysis was done using SPSS, version 28 (IBM, New York, USA). All variables were tested for normal distribution using a Shapiro-Wilk test. Two-sided paired t-test was used to detect statistically significant differences between methods. To test agreement between gait parameters from IMU’s and the AK the convergent validity with correlations via Spearman’s rank correlation coefficient (rsp) or Pearson product-moment correlation coefficient were calculated. Additionally, a Bland-Altman plot of the overall stride lengths and stride times displays the spatial and temporal agreement of both methods with the limits of agreement (LoA) for error-estimation of future measurements [31]. Tests between systems were calculated over all participants. The level of significance was set to $\alpha \le0.05$.

## III. Results

### A. Sample Size and Exclusions

Of the total 59 we excluded 5 subjects (n=54) due to missing data which was caused by technical problems with the treadmill belt leading to a premature termination of the test. Data of 34 female (62.9%) and 20 male (37.1%) participants were analyzed. Table 1 describes the characteristics of the participants, included for analyses.

**TABLE 1:** Anthropometric and Mobility Related Characteristics as Mean or Proportion Over All Participants (N=54). Walking Speed on the Treadmill was Assessed as Preferred Walking Speed. FES-I = Falls Efficacy Scale International

Age [years] mean±SD	76.8 ±4.7
(min-max)	(70–87)
(min-max)	(70-87)
Height [m] mean±SD	1.70 ±0.09
(min-max)	(1.52–1.85)
Weight [kg] mean±SD	74.3 ±12.8
(min-max)	(48.0–108.4)
BMI [kg $\cdot$ m^−2^] mean±SD	25.5±3.2
(min-max)	(19.7–32.3)
Treadmill walking speed [ $\text{m} \cdot \text{s}^{-1}$] mean±SD	0.85±0.18
(min-max)	(0.3–1.16)
FES-I [score 16–64] mean±SD	20.9±4.65
(min-max)	(16–35)
History of Falls in the last 12 months n (%)	26 (48,1%)
Glasses n (%)	51 (94.4%)
Hearing Aid n (%)	25 (47.2%)
Knee Joint Replacement n (%)	6(11.1%)
Hip Joint Replacement n (%)	9 (16.7%)

### B. Gait Parameters

Gait parameters from both systems are distributed normally. Table 2 shows the means of the calculated gait parameters and statistical differences between both methods. Only the cadence measured from AK and IMU were significantly different (p=0.005).

**TABLE 2:** Comparison of Gait Parameters, Measured With AK and IMU-System, With Mean Values ± Standard Deviation and Range (min-max) During Gait Analysis on a Treadmill

Parameter	AK mean±SD (min-max)	IMU mean±SD (min-max)	p-values
Cadence [steps $\cdot$ min^−1^]	107.6±9.3 (90-127)	105.5±9.5 (84-139)	0.005*
Stride time [s]	1.11±0.1 (0.93-1.32)	1.11±0.23 (0.83-1.36)	0.616
Single support time [s]	0.35±0.08 (0.29-0.42)	0.35±0.04 (0.25-0.45)	0.826
Double support time [s]	0.21±0.07 (0.15-0.30)	0.21±0.07 (0.14-0.45)	0.081
Stride length [cm]	96.0±16.8 (32.9-139.5)	95.3±19.4 (24.9-137.9)	0.143
Arm swing right [deg]	24.7±11.5 (8.0-62.7)	25.5±10.6 (7.4-71.8)	0.732
Arm swing left [deg]	31.8±11.3 (12.8-76.0)	28.0±10.5 (7.3-80.8)	0.075
Step width [cm]	15.6±2.9 (10.7-22.11)	X	X
MoS ML [cm]	9.8±3.34 (4.1-15.2)	X	X
MoS AP [cm]	6.5±5.4 (1.3-13.5)	X	X

^*^ =Significant difference from IMU (p<0.05), AK=Azure Kinect, IMU=Inertial Measurement Unit Diff=difference between IMU and AK; MoS=Margin of stability, ML=mediolaterai. AP=antepostenor; X=Not applicable

### C. Convergent Validity of the Systems

**FIGURE 2.**
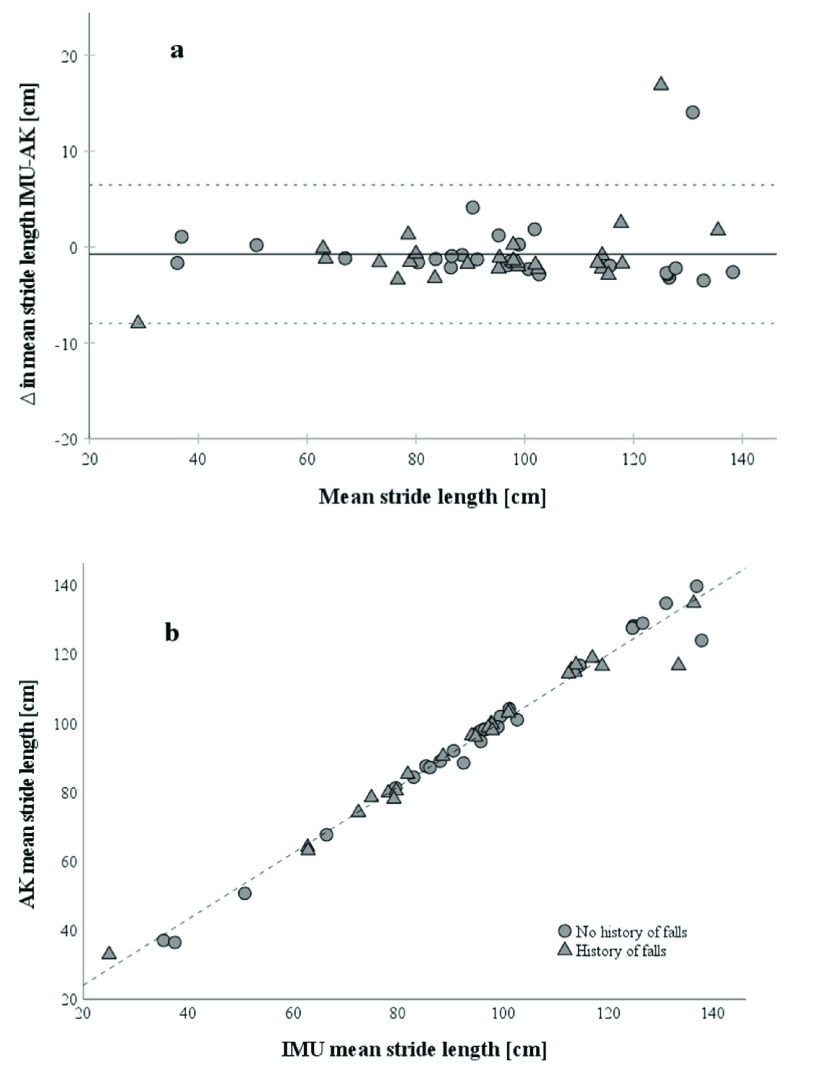
(a) Bland-Altman Plot of mean stride lengths (n=54) between IMU and AK. $\Delta $ as IMU stride length – AK stride length. The solid line represents the mean of $\Delta $ in mean stride lengths. Participants with a history of falls are marked as triangles, and participants without a history of falls as circles. Dotted lines represent the limits of agreement as upper and lower doubled standard deviation of the mean stride lengths between methods of measurement. (b) Correlation of stride lengths from IMU and AK with a Pearson correlation coefficient of r=0.979.

**FIGURE 3.**
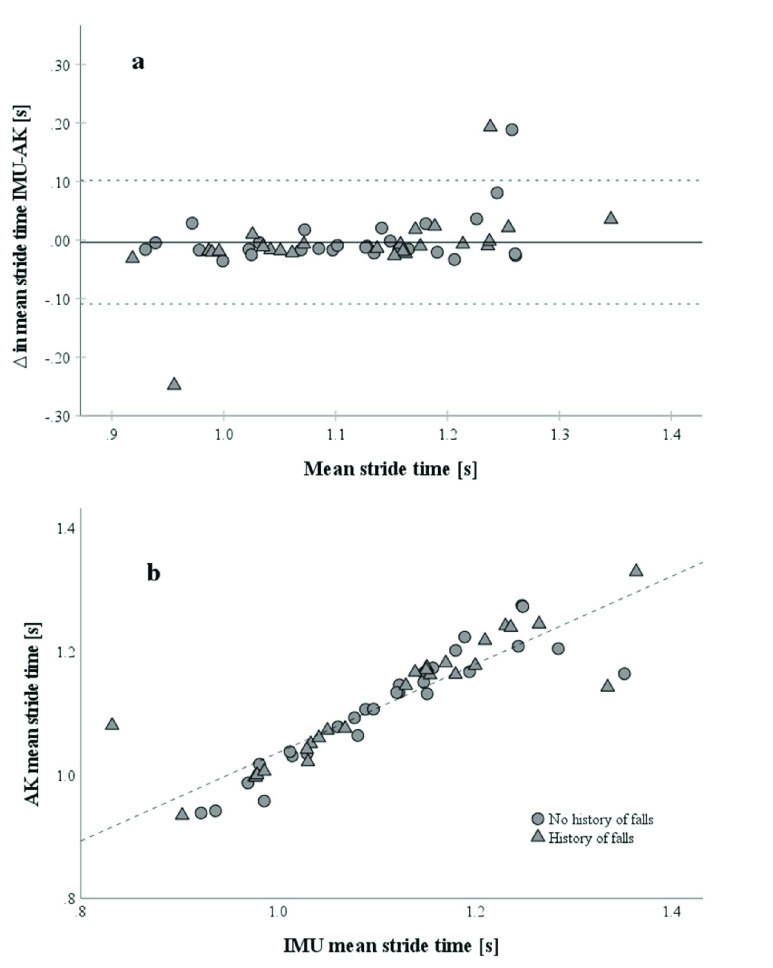
(a): Bland-Altman Plot of mean stride times (n=54) between IMU and AK. $\Delta $ as IMU stride time – AK stride time. The solid line represents the mean of $\Delta $ in mean stride times. Participants with a history of falls are marked as triangles, and participants without a history of falls as circles. Dotted lines represent the limits of agreement as upper and lower doubled standard deviation of the $\Delta $ in mean stride times between methods of measurement. (b) Correlation of stride times from IMU and AK with a Pearson correlation coefficient of r=0.914.

For convergent validity of IMU and AK, the Pearson correlation coefficients for stride length (r=.992, $\text{p} < 0.001$) (Fig. 2b), stride time (r=.914, $\text{p} < 0.001$) (Fig. 3b), single support time (r=.775, $\text{p} < 0.001$) and double support time (r=.767, $\text{p} < 0.001$) between the two systems are high. Figure 2b shows an almost linear relationship between IMU and AK stride lengths and Figure 3b depicts a strong relationship between IMU and AK stride times. Correlations between IMU and AK for the arm swing are low to moderate: Arm swing left (r=.556, $\text{p} < 0.001$), arm swing right (r=.385, $\text{p} < 0.001$) (Fig. 7b and 8b in Suppl. Material).

### D. Bland-Altman Plot for Agreement of Methods

Bland-Altman plots showed moderate agreement in spatial and temporal parameters. Difference in mean stride length between IMU and AK is −0.74 ± 3.68 cm with LoAs of −7.96 cm to 6.47 cm and 2 out of 54 (3.7%) data points outside of the LoA (Fig. 2a). And difference in mean stride time between IMU and AK is −3.7±54 ms with LoAs of −109 ms to 102 ms and 3 of 54 (4.7%) data points outside the LoA (Fig. 3a).

## IV. Discussion

### A. Spatiotemporal Gait Parameters

Spatiotemporal gait parameters from AK highly correlated with values of IMU (Fig. 2b and Fig. 3b). The Bland-Altman Plots for stride length and stride time showed a moderate agreement between the two systems with an estimated error of up to 7.21 cm and 105 ms, respectively (Fig. 2a and Fig. 3a). The outliers from stride length and stride time were the same subjects but a control of the data and comparison of the videos to other subjects did not reveal any abnormality. Overall, the graphs do not indicate a systematic difference in agreement for the two systems, between the groups. Regarding the other gait parameters, single and double support times have a lower convergent validity and agreement between IMU and AK (Suppl. Material, Fig. 5 and 6). Single support times are typically between 390 ms and 440 ms and double support times between 290 and 360 ms in comparable older adults from 70 to 85+ years [32]. With an AK sample rate of 33 ms, the relative error per sample is too high to detect small differences between groups or in longitudinal monitoring of subjects in single- and double support time. This could be explained by the calculation of the single and double support times based not only on the initial contact, as the other spatiotemporal parameters, but also on the toe-off, which has a higher error itself with the used algorithms for AK (mean error of −26 ± 22 ms for initial contact and −88 ± 34 ms for toe-off in comparison to marker-based systems [6]). This is also indicated by the lower correlations in single- and double support times between AK and IMU in relation to the correlations of stride length and stride time. Agreeing with Albert and colleagues [6] that spatiotemporal parameters calculated from AK data, relying only on the initial contact are suitable for treadmill gait analysis. Higher step width in comparison to normative values from Hollmann et al. [24] were observed, but step width from AK was calculated from the midpoint of the ankle joint and not from the inner boundaries of the foot which are commonly used to calculate step width [32]. Step width was comparable to AK gait parameters from Albert et al. [6] (17±3 cm).

### B. Margin of Stability

The margin of stability in mediolateral direction with 9.8±3.3 cm appeared larger compared to 6.1±1.3 cm for healthy older adults [22] and 8.89±1.2 cm for healthy younger adults [33] from marker-based motion capture but also had a higher variability. Margin of stability in anteposterior direction with 6.5±5.4 cm was smaller compared to 9.38±2.86 cm in healthy younger adults and had also a higher variability [33]. AK has a lower spatial and temporal resolution as marker-based motion capture, which likely contributes to a higher variability but also calculates the midpoint of the pelvis as the center of mass instead of a marker attached to lower lumbar level or sacrum (marker-based motion capture) which likely results in a systematical difference in anteposterior margin of stability.

### C. Arm Swing

For the arm swing amplitude algorithm from IMU data Warmerdam et al. [13] report a small systematic error of 0.9-1.1 degree, while the average position error of wrists from AK is about 25 mm and up to 30 mm [6]. Therefore, if the error of 30 mm applies to the maximal forward and backward position of the arm, the summarized error of one swing would be as high as 60 mm. The convergent validity and agreement of methods for arm swing amplitude are relatively low (Suppl. material Fig. 7 and 8). Arm swing amplitude results also indicated a significant difference between the left and right arm for both systems with a higher difference in AK. Excessive arm swing amplitude on one side is common [34], and due to slow sample rate, the cameras are likely to perform poorly in faster movements (e.g. bigger arm swing at same stride time). We found no comparable studies concerning this problem. Decreased arm swing amplitude and symmetry in older people have a negative effect on gait [35]. Interpretation of this arm swing data from AK should be done with caution regarding a possible performance issue of AK with arm movements.

### D. Framework for Azure Kinect in Gait Analysis

As a result of the low temporal resolution of AK compared to the temporal dimension of single support times, double support times and arm swing amplitude, substantial differences in these parameters can be between to data frames (33 ms). Therefore, no conclusions should be drawn from these data. The general use of this type of depth camera for adequate parameters like stride length and stride time requires still some considerations. We would advise to clear the capturing area of persons during measurement to limit the pose estimation to the patient and avoid therefore common and sudden switching of the body-designation in the pose estimation algorithm. The Microsoft SDK provides basic interaction with the camera, for advanced use, a customized software is recommended. At the time of the study a certain type of high-performance graphics-processor is required for body tracking of depth videos [5]. The error of AK could have its origin in the depth-images, the artificial intelligence algorithm of the body tracking and the gait parameter algorithm. Also, one has to keep in mind, that these results are only valid for averaged values over two minutes and the possible errors for step to step analysis are higher. The use of a single depth-camera for gait analysis is preferable for treadmill walking and limited in over ground walking, because of the restriction of a capture-area [15] and diverging accuracy over different distances between the person and the camera [23]. Ongoing updates for tracking algorithms, and the fast-paced development of new cameras are promising that accuracy can be optimized and cameras can operate at a higher sample rate.

### E. Translation Into Clinical Practice

Given the aforementioned adaptations, paired with software for automatic gait analysis, the implementation for institutions, that already use treadmills as a tool for training, diagnosis- or rehabilitation, is reduced to the setup of the camera (e.g. via tripod) in front of the treadmill and the operation of a connected computer. Furthermore, the use of depth-cameras can be simplified with intuitive user interfaces for clinicians and therapists. As a result of a marker-less principle of AK, the patient does not need to be prepared with sensors or markers before measurement leading possibly to better acceptance in older adults and more frequent analysis. In summary, this simple infrared-technology paired with AI could allow for a quick to perform gait analysis in a controlled environment without the application of body-worn sensors or markers on the patient.

### F. Limitations

Restricted by the study setting, motion capturing with AK was not compared with a marker-based motion capture system as gold-standard. However problems with interferences between the infrared emitters of marker-based motion capture and AK resulting in large areas without depth-information were reported [15]. Rather, an inertial measurement system was used for comparison which represents a validated and available system for clinical settings [36]. Furthermore, systems were not synchronized for step by step analysis and instead the same time interval, the last 120 seconds of walking, was analyzed. An unknown potential source for error besides the depth measurement is also the implemented artificial intelligence of the Body racking SDK. Additionally, it is possible that white or black clothes as well as the surrounding infrared light can affect the pose estimation performance [37]. We did not control these circumstances.

## V. Conclusion

Gait parameters from a depth-camera, averaged over 120 seconds of treadmill walking in older adults at risk of falling, have a higher convergent validity to IMU-data when based on initial contact instead on initial- and toe-off e.g. stride length and step time instead of single- and double support time. Due to the good agreement of methods in stride length and stride time the results of our study promote the use of depth-cameras in treadmill gait analysis of older people with and without increased risk of falling as an alternative to the use of IMUs. Furthermore, depth-cameras have the potential to allow measurement of stability-relevant metrics [38], without interfering with the patient’s personal space, and are available at relatively low cost. However, this method is to be further validated and body tracking, spatial resolution as well as low framerate are technical problems to be considered and solved. The next steps for gait analysis in people at risk of falling should also include the technical difficulties to assess gait parameters in balance challenging situations, for example from mechanical treadmill perturbations, which are able to highlight balance insufficiencies [39].

## Supplementary Materials

Supplementary materials
