# Supplementary material for: Depth-Imaging for Gait Analysis on a Treadmill in Older Adults at Risk of Falling
Source: IEEE J Transl Eng Health Med. 2023 May 19;11:479–86. doi: 10.1109/JTEHM.2023.3277890 (PMC10561749; doi:10.1109/JTEHM.2023.3277890)
Supplement: Supplementary materials [file supp1-3277890.docx]

# Supplementary


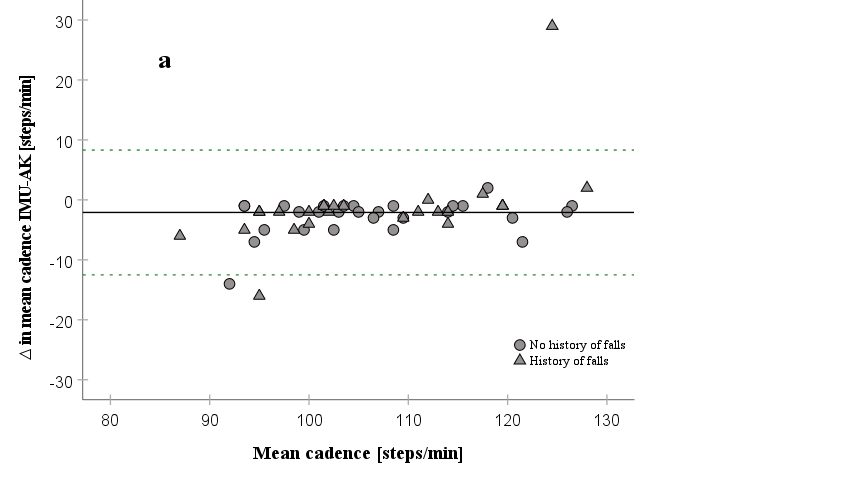

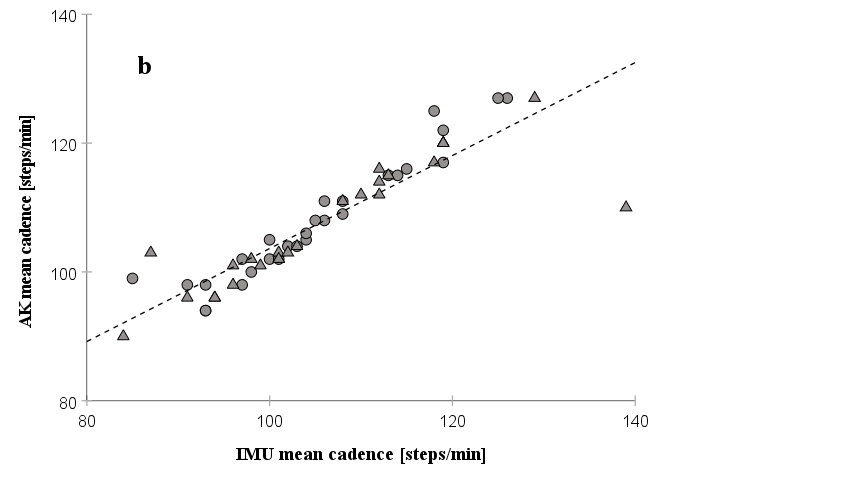


Fig. 4: (a) Bland-Altman Plot of mean cadence (n=54) between IMU and AK. ∆ as IMU cadence – AK cadence. The solid line represents the mean of ∆ in mean cadence. Dotted lines represent the limits of agreement as upper and lower doubled standard deviation of the mean cadence between methods of measurement. (b) Correlation of cadence from IMU and AK of all groups with a Pearson correlation coefficient of r=0.799.


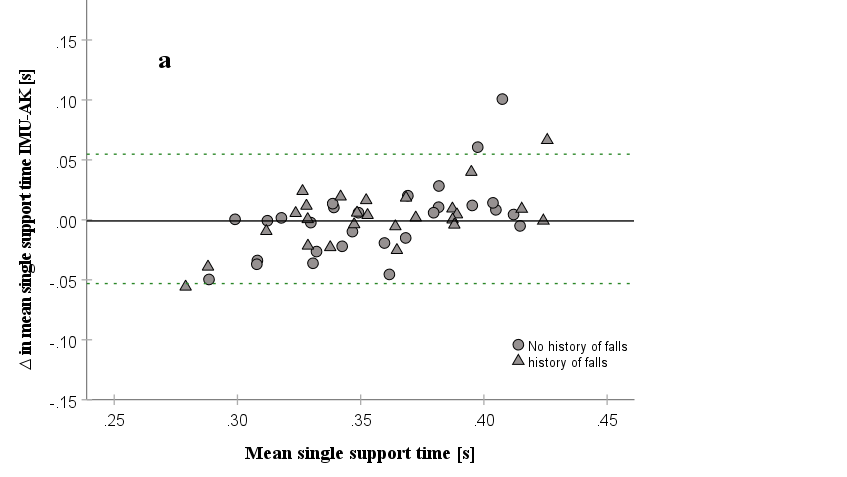

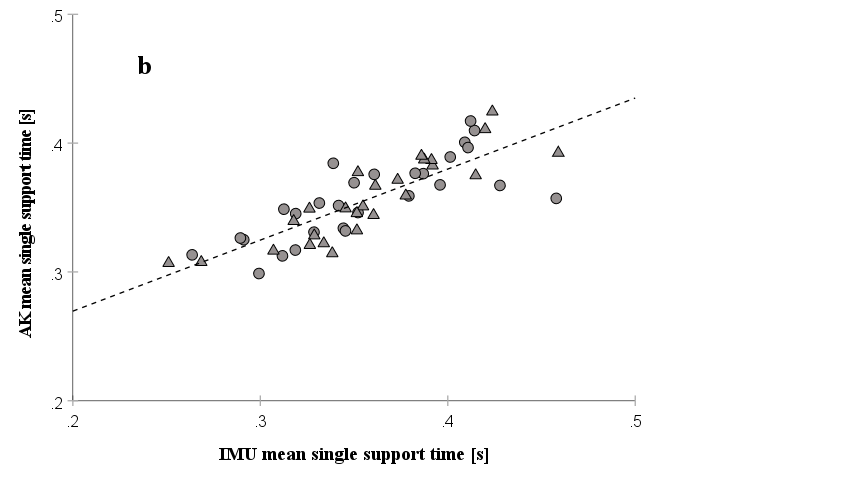


Fig. 5: (a) Bland-Altman Plot of mean single support time (n=54) between IMU and AK. ∆ as IMU single support time – AK single support time. The solid line represents the mean of ∆ in mean single support time. Dotted lines represent the limits of agreement as upper and lower doubled standard deviation of the mean single support time between methods of measurement. (b) Correlation of single support time from IMU and AK of all groups with a Pearson correlation coefficient of r=0.676.


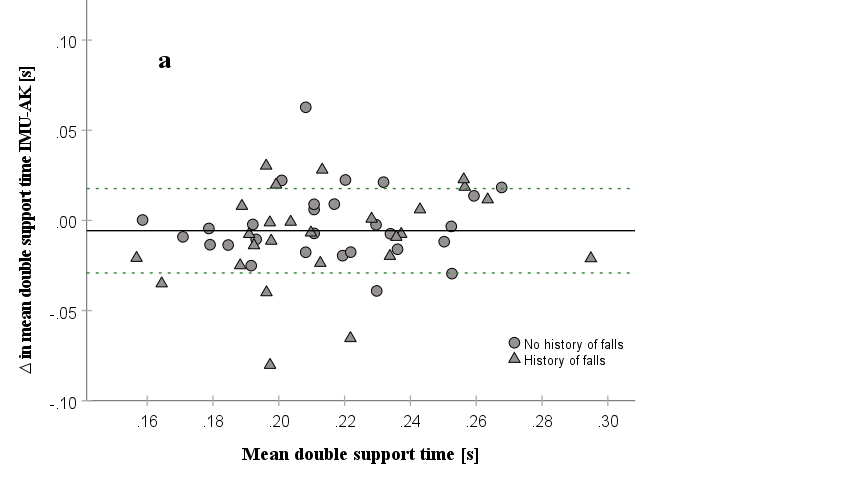

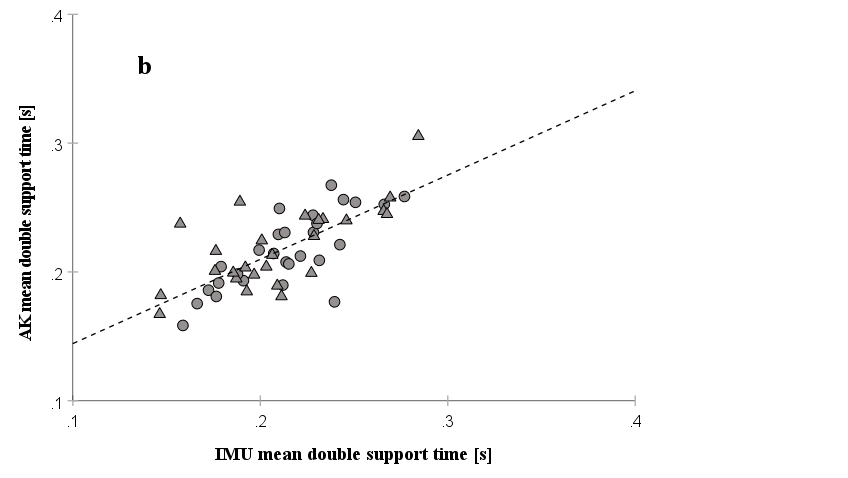


Fig. 6: (a) Bland-Altman Plot of mean double support time (n=54) between IMU and AK. ∆ as IMU double support time – AK double support time. The solid line represents the mean of ∆ in mean double support time. Dotted lines represent the limits of agreement as upper and lower doubled standard deviation of the mean double support time between methods of measurement. (b) Correlation of double support time from IMU and AK of all groups with a Pearson correlation coefficient of r=0.532.


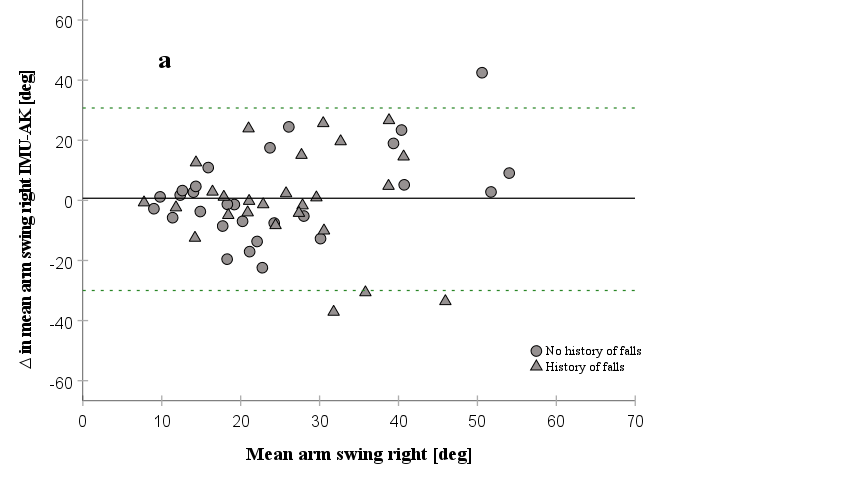

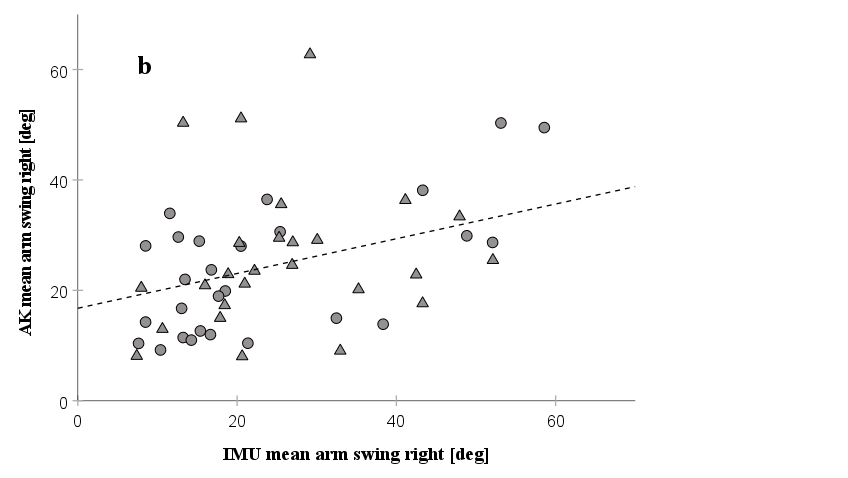


Fig. 7: (a) Bland-Altman Plot of mean arm swing amplitude right (n=54) between IMU and AK. ∆ as IMU arm swing amplitude right – AK arm swing amplitude right. The solid line represents the mean of ∆ in mean arm swing amplitude right. Dotted lines represent the limits of agreement as upper and lower doubled standard deviation of the mean arm swing amplitude right between methods of measurement. (b) Correlation of arm swing amplitude right from IMU and AK of all groups with a Pearson correlation coefficient of r=0.148.


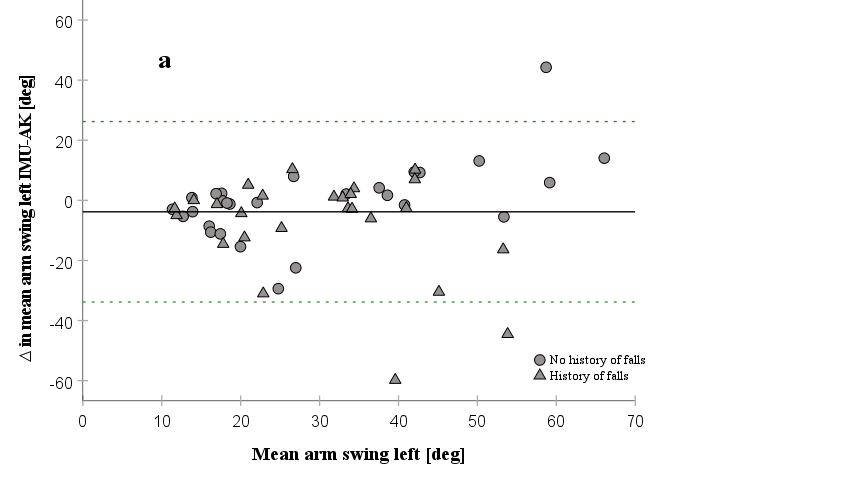

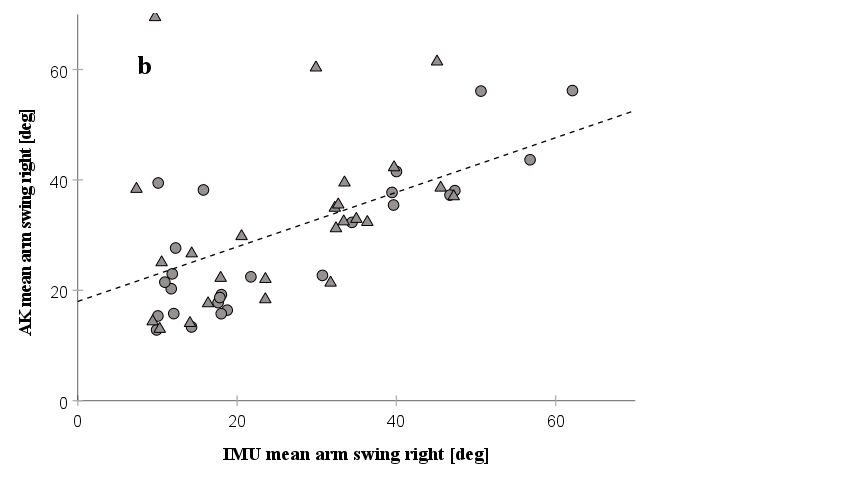


Fig. 8: (a) Bland-Altman Plot of mean arm swing amplitude left (n=54) between IMU and AK. ∆ as IMU arm swing amplitude left – AK arm swing amplitude left. The solid line represents the mean of ∆ in mean arm swing amplitude left. Dotted lines represent the limits of agreement as upper and lower doubled standard deviation of the mean arm swing amplitude left between methods of measurement. (b) Correlation of arm swing amplitude left from IMU and AK of all groups with a Pearson correlation coefficient of r=0.309.
